# Association between Fat Mass- and Obesity- Associated (FTO) Gene Polymorphism and Polycystic Ovary Syndrome: A Meta-Analysis

**DOI:** 10.1371/journal.pone.0086972

**Published:** 2014-01-22

**Authors:** Xianli Cai, Chibo Liu, Sihua Mou

**Affiliations:** 1 Department of Gynaecology, Taizhou Municipal Hospital, Taizhou, China; 2 Department of Clinical Laboratory, Taizhou Municipal Hospital, Taizhou, China; Imperial College London, United Kingdom

## Abstract

**Aims:**

Many studies have investigated the relationship between *FTO* gene polymorphism and polycystic ovary syndrome (PCOS) susceptibility but revealed mixed results. In this study, we aimed to perform a meta-analysis to clarify this association.

**Methods:**

Published literature from PubMed, Embase and CNKI was retrieved. Meta-analysis was performed to calculate pooled odds ratio (OR) with 95% confidence interval (CI) using the random- or fix- effects model.

**Results:**

A total of 5 studies (4778 cases and 4272 controls) were included in our meta-analysis. The results suggested that *FTO* rs9939609 polymorphism (or its proxy) was marginally associated with PCOS risk after adjustment for body mass index (BMI) (OR = 1.26; 95%CI: 1.02–1.55). However, the marginal association was not stable after sensitivity analysis. In the subgroup analysis by ethnicity, the association was significant in East Asians (OR = 1.43, 95%CI = 1.30–1.59) but not in Caucasians (OR = 1.04, 95%CI = 0.85–1.29).

**Conclusions:**

Our present meta-analysis indicated that *FTO* rs9939609 polymorphism (or its proxy) might not be associated with risk of PCOS in overall population. However, in East Asians, there might be a direct association between *FTO* variant and PCOS risk, which is independent of BMI (adiposity).

## Introduction

Polycystic ovary syndrome (PCOS) is a common endocrine disorder affecting 5–7% of women of childbearing age [1]–[3]. It is characterized by hirsutism, anovulation, clinical and/or biochemical hyperandrogenism, and polycystic ovarian morphology on ultrasound [4]. A large number of women with PCOS also exhibit insulin resistance, β-cell dysfunction, impaired glucose tolerance and/or type 2 diabetes [5]. It has been reported that about half of PCOS women are overweight or obese [6] and obesity plays an important role in the etiology of PCOS [7]–[9]. Given the high prevalence of obesity in PCOS, both diseases may share similar genetic background [9]. PCOS is a highly complex and heterogeneous disorder. So far, the pathogenesis of PCOS is still incompletely understood, it may be affected by the environmental or genetic factors, or their interactions.

The fat mass and obesity associated gene (*FTO*), located in chromosome 16q12.2 and expressed in the adipose tissue and the brain and muscles, was proved to associate with body mass index and obesity [10]. The *FTO* protein regulates energy metabolism to increase the obesity risk [11].

Up to date, several studies have been performed to investigate the association between *FTO* gene polymorphism and the PCOS susceptibility. Some studies reported that *FTO* rs9939609 polymorphism (or its proxies) was positively associated with PCOS [12]–[16], while others showed no significant association [17]–[19]. The differences may be due to many factors, including the insufficient statistical power and whether adjustment for body mass index (BMI) in each individual study.

In order to increase the statistical power of individual studies, we performed a meta-analysis to clarify the relationship between *FTO* gene polymorphism and the risk of PCOS across different ethnic populations.

## Methods

### Literature and search strategy

We searched the PubMed, Embase and CNKI databases from 2007 to 2013 since *FTO* gene rs9939069 polymorphism and its association with obesity was first reported in 2007. The search strategy to identify all potential studies involved the use of the following key words: (PCOS OR polycystic ovary syndrome) and (fat mass and obesity associated gene OR *FTO*). The publication language was restricted to English or Chinese. The reference lists of retrieved articles were hand-searched. If more than one article were published using the same case series, only the study with largest sample size was included.

### Inclusion criteria and data extraction

A study was included in the meta-analysis only if it met all the following inclusion criteria: (1) evaluated the association of any of the *FTO* polymorphisms with PCOS; (2) used case-control or cohort design; (3) provided OR with 95%CI or sufficient data for calculation of the estimate. The following information was extracted from each study: (1) name of the first author; (2) year of publication; (3) origin of country; (4) ethnicity; (5) number of cases and controls; (6) mean age and BMI of subjects; (7) OR with 95% CI under an additive model; (8) single nucleotide polymorphisms (SNPs); and (9) *P* value for Hardy-Weinberg equilibrium (HWE) test in controls. Two authors independently assessed the articles for compliance with the inclusion/exclusion criteria, and resolved discrepancies by group discussion until reaching a consistent decision.

### Statistical analysis

The association of *FTO* polymorphism with PCOS was estimated by calculating the pooled OR and 95% CI. The significance of the pooled OR was determined by Z test (*P*<0.05 was considered statistically significant). The Q test was performed to evaluate to the degree of between-study heterogeneity. A random-effects (DerSimonian–Laird) [20] or fixed-effects (Mantel–Haenszel) [21] model was used to calculate the pooled OR in the presence (*P*≤0.10) or absence (*P*>0.10) of heterogeneity, respectively. Publication bias was assessed by Begg's test[22] and Egger's test [23] (*P*<0.05 was considered statistically significant). To evaluate the stability of the results, we performed a sensitivity analysis by removing one study at a time. Data were analyzed by STATA version 11.0 (StataCorp LP, College Station, TX, USA).

## Results

### Characteristics of the included studies

**Figure 1:**
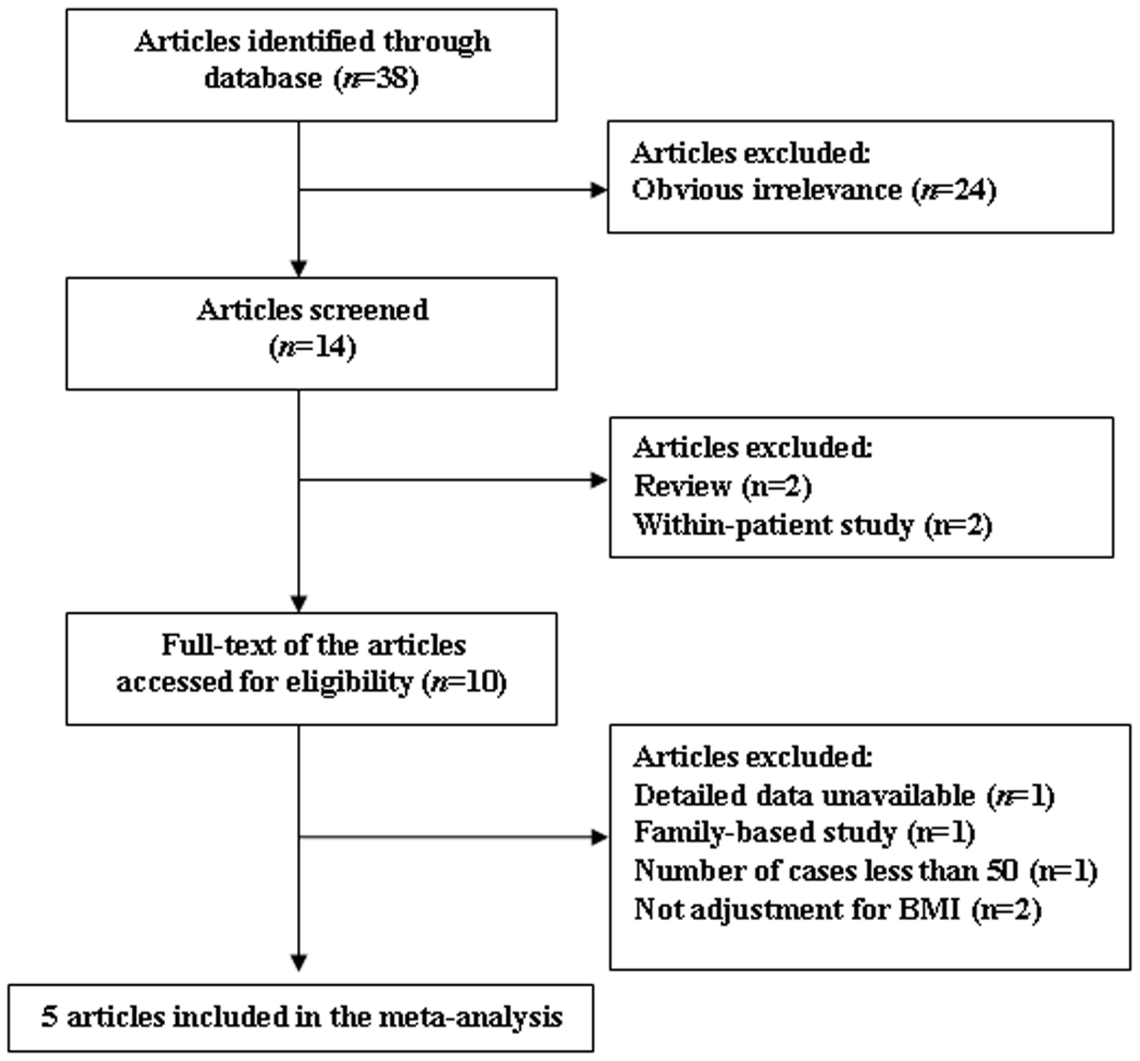
Flow chart of article selection for meta-analysis.

The literature search identified a total of 38 potentially relevant articles. Of these, 24 were excluded after reading the title or abstract because of obvious irrelevance, 13 potentially relevant articles remained for further full-text evaluation. Of these, 2 articles [9], [24] were excluded because they were within-patient study. One article [25] was excluded because of lacking data for calculation of OR with 95% CI. One article [17] was excluded because it was a family-based study. 2 articles [28], [29] were excluded as they were reviews. One article was excluded since the number of cases was less than 50 [13]. Two studies were excluded as both did not adjust for BMI which is one of the main confounding factors for the association [14], [15]. At last, a total of 5 studies (4778 cases and 4272 controls) were included in our meta-analysis. A flow chart describing the process of study inclusion/exclusion is displayed as Figure 1.

All included studies used the Rotterdam criteria [26] to diagnose PCOS except for the study by Saxena et al. [5] used the NIH criteria [27]. The genotypes in the controls of all included studies were in Hardy-Weinberg equilibrium. Of these studies, there were 3 studies for rs9939609 [5], [12], [16], one study [19] for rs1421085, one study [18] for rs8050136. Since rs9939609 was in high LD with rs1421085, rs8050136 (all r^2^>0.90), we selected the rs9939609 polymorphism as the representative. Two ethnicities were involved in our 5 studies: 2 studies [5], [18] were on Caucasian population, 3 studies [12], [16], [19] were on East Asian population. ORs and 95%CIs for the majority of studies were generated under an additive genetic model, so we calculated the summary estimate under this model. Characteristics of the included studies are shown in Table 1.

**Table 1:** Characteristics of studies included in meta-analysis.

Study	publication year	Country	Ethnicity	*FTO* SNP	Sample size		Mean age(years)		Mean BMI(kg/m^2^)		OR	95%CI	*P*_HWE_ a
					cases	controls	cases	controls	cases	controls			
Yan et al. [12]	2009	China	East Asian	rs9939609	215	227	21.7	27.5	28.0	20.8	1.39	0.76–2.52	0.36
Hatziagelaki et al.[18]	2012	Germany	Caucasian	rs8050136	62	105	26.0	27.0	27.3	26.6	1.24	0.58–2.64	0.49
Kim et al. [19]	2012	Korea	East Asian	rs1421085	377	386	28.5	28.5	22.2	20.1	1.13	0.47–2.68	0.46
Saxena et al.[5]	2012	USA	Caucasian	rs9939609	525	472	28.4	28.9	30.8	23.5	1.03	0.83–1.28	0.69
Li et al. [16]	2013	China	East Asian	rs9939609	3599	3082	28.4	31.3	24.8	22.7	1.44	1.30–1.60	0.74

OR =  odds ratio, CI =  confidence interval, SNP = single nucleotide polymorphism.

a *P* value for Hardy–Weinberg equilibrium test (HWE) in controls.

### Meta-analysis results

**Figure 2:**
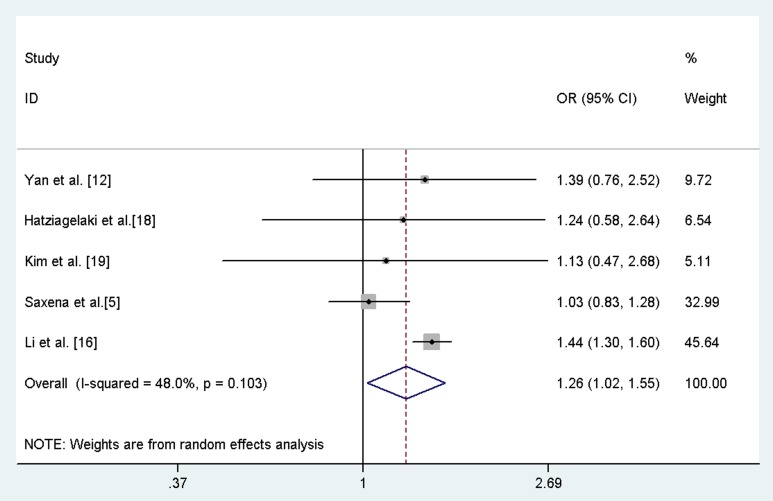
Forest plot of meta-analysis of the association between rs9939609 (or its proxy) and PCOS after adjusted for BMI under an additive model.

The overall result showed a marginal association between *FTO* rs9939609 polymorphism (or its proxies) and PCOS risk under an additive model after adjustment for BMI (OR = 1.26; 95%CI: 1.02–1.55; Figure 2), with modest evidence of between-study heterogeneity (*I* ^2^ = 48.0%; *p* = 0.103). Since the study by Li et al. [16] made up the largest proportion (45.6%) of all included studies, we excluded this study and re-calculated the pooled estimate. The result suggested that the marginal association completed disappeared (OR = 1.08, 95%CI = 0.89–1.31).

In the subgroup analysis by ethnicity, the association after adjustment for BMI was significant in East Asians (OR = 1.43, 95%CI = 1.30–1.59) but not in Caucasians (OR = 1.04, 95%CI = 0.85–1.29).

### Sensitivity analysis

Sensitivity analysis was performed by removing one study at a time. The marginal association after adjustment for BMI was not stable, with ORs and 95%CIs ranging from 1.08 (0.89–1.31) to 1.43 (1.29–1.58).

### Publication bias

The results suggested no publication bias after adjustment for BMI (Egger's test: *p* = 0.462 and Begg's test: *p* = 0.479).

## Discussion

The present meta-analysis suggested that the *FTO* gene polymorphism was marginally associated with an increased risk of PCOS after adjustment for BMI. However, the marginal association was not stable in the sensitivity analysis. In the subgroup analysis, the association was significant in East Asians but not in Caucasians, suggesting the different ethnic effect on the association.

Up to now, the mechanism underlying the association of the *FTO* gene polymorphism with PCOS risk remains unclear. One recent meta-analysis suggested that the effect of *FTO* polymorphism on obesity-related traits in PCOS seems to be more than two times greater than the effect found in large population-based studies [28]. Our meta-analysis showed that the association with PCOS disappeared after adjustment for BMI, indicating that the association between *FTO* gene rs9939609 polymorphism with PCOS susceptibility is probably by its effect on adiposity. This indirect mechanism was supported by the evidence that the effect estimates got far weaker when limited to the leaner PCOS patients in case-control study [15]. The effect of *FTO* gene on obesity is mainly by increasing energy intake [30] and reducing satiety sensation[31]. In addition, *FTO* gene variation might influence the baseline lipid oxidation to increase the obesity risk in PCOS [25].

At present, it has been reported that *FTO* gene influence PCOS mainly via the association with obesity or obesity-related parameters[9], [12], [14], [15], [24], [31], such as BMI, body weight, fat mass and some other metabolism-related traits[9], [14], [24], [32], such as insulin resistance, impaired fasting glucose, glucose intolerance. It has been reported that the impact of the *FTO* gene polymorphism on the metabolic parameters could be due to its effect on BMI [33]. The effect of the *FTO* gene on PCOS is possibly related to its genetic interaction with other susceptibility genes, and they combined to create the polygenic background of PCOS [32]. Another direct mechanism might be that the inverse correlation between fat mass and sex hormone binding globulin (SHBG) levels in women may result in lower SHBG levels in those with *FTO* risk allele, which in turn would lead to higher free testosterone [34]. In regards to indirect mechanism, insulin resistance and hyperinsulinaemia may increase in subjects with *FTO* risk allele, then resulting in hyperandrogenaemia throuh ovarian co-gonadotrophic effects [35].

The current meta-analysis has some limitations in spite of several advantages compared to individual studies. First, some potential confounding factors or modifiers were not controlled for. For instance, two included studies did not provide BMI-adjusted data [14], [15]. Second, the gene-gene/gene-environment interaction was not addressed in the current meta-analysis since no related data was provided by the original publications. Third, the underlying genetics may be quite different in subjects with different sub-phenotypes of PCOS. The criteria of Rotterdam Revised 2003 stated that subjects fulfilled the following 2 out of 3 items were diagnosed as PCOS: (i) oligomenorrhea or amenorrhea for at least 6 months; (ii) clinical and/or biochemical signs of hyperandrogenism; (iii) polycystic ovaries (the presence of 12 or more follicles in each ovary measuring 2–9 mm in diameter) and/or increased ovarian volume (>10 ml) [26]. However, nearly all the studies did not specify the number of subjects for each subset of phenotypes. Thus, the majority of included studies did not provide the data for sub-phenotypes of PCOS versus controls. Then, we were unable to examine the association between *FTO* variant and sub-phenotypes of PCOS.

In conclusions, our meta-analysis results indicated that *FTO* rs9939609 polymorphism (or its proxy) was not related to PCOS susceptibility after adjustment for BMI. However, in East Asians, there might be a direct association between *FTO* variant and PCOS risk since the association was significant after adjustment for BMI. Further studies should be conducted to explore the association of *FTO* gene polymorphism with PCOS.
